# Lower serum clusterin levels in patients with erosive hand osteoarthritis are associated with more pain

**DOI:** 10.1186/s12891-018-2179-3

**Published:** 2018-07-27

**Authors:** Tereza Kropáčková, Olga Šléglová, Olga Růžičková, Jiří Vencovský, Karel Pavelka, Ladislav Šenolt

**Affiliations:** 1 Institute of Rheumatology, Prague, Czech Republic; 2 Department of Rheumatology, 1st Faculty of Medicine, Charles University, Prague, Czech Republic

**Keywords:** Hand osteoarthritis, Erosive osteoarthritis, Clusterin

## Abstract

**Background:**

The aims of this study were to analyse the serum concentrations of clusterin (CLU) in patients with hand osteoarthritis (OA) and in healthy controls, to compare CLU levels between patients with erosive and non-erosive disease, and to examine the association of CLU levels with clinical and laboratory parameters.

**Methods:**

A total of 135 patients with hand OA (81 with erosive and 54 with non-erosive disease) and 53 healthy individuals were included in this study. All patients underwent clinical and hand joint ultrasound examination. The Australian/Canadian (AUSCAN) hand osteoarthritis index, algofunctional index and a visual analogue scale (VAS) for the measurement of pain were assessed. Serum levels of CLU were measured by an enzyme-linked immunosorbent assay (ELISA).

**Results:**

Serum levels of CLU were significantly lower in patients with hand OA than in control subjects (*p* < 0.0001). In addition, patients with erosive hand OA had significantly lower CLU levels than those with non-erosive disease (*p* = 0.044). Negative correlations between CLU levels and pain as assessed by the AUSCAN score and the VAS were found in patients with erosive hand OA (*r* = − 0.275; *p* = 0.013 and *r* = − 0.220; *p* = 0.049, respectively).

**Conclusion:**

The present study demonstrates that lower concentrations of CLU are found in hand OA patients than in healthy individuals, especially in those with erosive disease, and that CLU concentrations have a negative association with hand pain.

## Background

Osteoarthritis (OA) of the hands is a degenerative joint disease primarily affecting the interphalangeal and thumb base joints. Hand OA is common among the elderly, especially in women. It may cause pain and disability, and it negatively affects the patients’ quality of life [1, 2]. The erosive form of hand OA is defined radiographically by its subchondral erosion, cortical destruction and subsequent reparative changes, which may include bony ankylosis. Erosive OA typically has an abrupt onset and is accompanied by local inflammation and worse symptoms than non-erosive disease [3].

Clusterin (CLU), also known as apolipoprotein J, is a protein that is involved in a number of biological processes, including inflammation and apoptosis. CLU exists in several distinct isoforms that differ in their structure, function and localization. The predominant isoform, secretory clusterin (sCLU), is a heterodimeric glycoprotein that acts as a molecular chaperone [4] and exhibits anti-apoptotic and pro-survival activities [5]. Nuclear clusterin (nCLU) arises via an alternative splicing of the *CLU* gene leading to exclusion of exon II [6] and acts as a pro-apoptotic molecule [7]. Cellular forms of CLU are relatively rare, and their function is still poorly understood, but it does not appear that they affect the apoptotic pathway [8].

Clusterin is produced in many tissues, including articular cartilage and the synovium. Higher expression of CLU mRNA has been reported in early OA than in normal cartilage [9]. In advanced OA cartilage, CLU mRNA expression was reduced in comparison with that found in early OA. Based on these results, the authors proposed a potential role of CLU in the maintenance of articular cartilage. The upregulated expression of CLU in early OA might reflect an effort of chondrocytes to protect and repair the cartilage tissue while the downregulated CLU mRNA expression and consequently the loss of this protection in the advanced OA cartilage accompanies the final degenerative stage of the disease [9]. Fandridis et al. detected increased expression of sCLU mRNA in advanced OA compared with the expression of that in healthy cartilage. Moreover, they found higher serum sCLU levels in patients with advanced OA than in healthy individuals. Therefore, CLU could be suggested as a biomarker reflecting cartilage tissue changes [10].

CLU mRNA expression in synovial tissue is decreased in rheumatoid arthritis (RA) compared with its expression in OA or healthy tissue, but the protein levels in synovial fluid are equally present in RA and OA [11]. In cultured fibroblast-like synoviocytes (FLS), CLU inhibits nuclear factor (NF)-κB activation and modulates the expression of genes in the response to tumor necrosis factor (TNF)-α stimulation [11, 12]. Recently, sCLU has been shown to inhibit osteoclast proliferation and differentiation, and its protective role against bone erosions has been suggested [13].

The aims of this study were therefore to compare the serum levels of CLU between patients with hand OA and healthy subjects and between OA patients with erosive and non-erosive disease and to investigate the association of CLU levels with measures of disease severity.

## Methods

### Patients

A total of 135 patients with hand OA (81 with the erosive and 54 with the non-erosive form) and 53 healthy individuals were included in this study. The demographic and clinical characteristics of the subjects are summarized in Table 1. The exclusion criteria for all subjects were the presence of systemic inflammatory disease or cancer; the healthy controls showed no clinical signs of hand OA. All patients fulfilled the American College of Rheumatology (ACR) classification criteria for hand OA [14]; patients with erosive disease had at least one interphalangeal joint with radiographic signs of erosions. Ultrasound of all joints of both hands for the detection of osteophytes and the assessment of power Doppler (PD) and gray scale (GS) synovitis was performed by two ultrasonographers using Esaote Mylab 60 equipment (Esaote S.p.A., Genova, Italy) using a linear transducer with a 18 MHz frequency. Synovitis in the GS and PD were scored semiquantitatively (0–3) as described earlier [15]. The ultrasonographers were unaware of patient’s clinical examination and laboratory findings. Inter- and intra-observer reliability has recently been published with moderate to very good results [15]. The clinical examinations were performed by qualified rheumatologists. Pain, stiffness and function were assessed by Australian/Canadian (AUSCAN) hand osteoarthritis index [16]. Hand disability was further determined using an algofunctional index [17]. A visual analogue scale (VAS) was used for the assessment of pain. Radiographs of the knees and hips were evaluated for the presence of OA using the Kellgren-Lawrence system in all patients [18]. Written informed consent from each subject was obtained prior to enrolment, and the study was approved by the local ethics committee.

**Table 1:** Characteristics of patients with hand OA and control subjects

	OA patients (*n* = 135)	Erosive (*n* = 81)	Non-erosive (*n* = 54)	Controls (*n* = 53)
Age, years	66.3 ± 8.3	67.6 ± 8.6*	64.3 ± 7.3	64.6 ± 7.6
Sex, female/male	120/15	74/7	46/8	48/5
CRP, mg/l	3.2 ± 3.9	3.4 ± 4.1	2.9 ± 3.7	3.3 ± 5.1
BMI, kg/m^2^	27.2 ± 4.2	27.5 ± 4.5	26.8 ± 3.7	NA
Disease duration, years	4.4 ± 5.2	4.4 ± 5.1	4.5 ± 5.5	NA
AUSCAN	22.5 ± 10.5	23.9 ± 11.1*	20.3 ± 9.1	NA
AUSCAN - pain	8.4 ± 4.2	9.0 ± 4.4*	7.5 ± 3.8	NA
AUSCAN - stiffness	1.9 ± 0.9	2.0 ± 0.9	1.9 ± 0.9	NA
AUSCAN - function	12.0 ± 6.4	12.8 ± 6.8	10.9 ± 5.6	NA
Algofunctional index	18.6 ± 5.9	19.5 ± 6.4	17.2 ± 4.8	NA
VAS - pain, mm	44.2 ± 22.7	46.7 ± 24.0	40.3 ± 20.3	NA
US osteophytes, n	12.8 ± 5.1	14.0 ± 4.6**	11.0 ± 5.4	NA
GS synovitis (total)	7.5 ± 8.7	9.3 ± 9.2***	4.8 ± 7.2	NA
GS synovitis (joint count)	5.5 ± 6.4	6.5 ± 6.6***	3.9 ± 5.8	NA
PD synovitis (total)	2.0 ± 2.7	2.5 ± 3.1*	1.3 ± 1.9	NA
PD synovitis (joint count)	1.7 ± 2.1	2.1 ± 2.3**	1.1 ± 1.6	NA
Knee OA, n (%)	59 (44)	35 (43)	24 (44)	NA
Hip OA, n (%)	40 (30)	27 (33)	13 (24)	NA
Knee and hip OA, n (%)	28 (21)	18 (22)	10 (19)	NA

The data are presented as the mean ± the standard deviation. * *p* < 0.05, ** *p* < 0.01, *** *p* < 0.001 compared with non-erosive. (*AUSCAN* Australian/Canadian, *BMI* body mass index, *CRP* C-reactive protein, *GS* gray scale, *OA* osteoarthritis, *PD* power Doppler, *US* ultrasound, *VAS* visual analogue scale)

### Laboratory measurements

Peripheral blood samples were obtained from all individuals and immediately centrifuged. The serum samples were stored at − 80 °C until their analysis. C-reactive protein (CRP) levels were measured turbidimetrically using the Beckman Coulter AU system (Beckman Coulter, Brea, CA, USA). The serum CLU concentrations were analysed by an enzyme-linked immunosorbent assay (ELISA) in compliance with the manufacturer’s instructions (BioVendor, Brno, Czech Republic). The samples from the patients and the healthy individuals were analysed together in each ELISA plate. As claimed by the manufacturer, the antibodies used in this ELISA are specific for human CLU, the assay detection limit is 5 ng/ml and the detection range is 5–160 ng/ml. The manufacturer’s stated intra-assay and inter-assay coefficients of the variations are 6.2 and 7.8%, respectively. The final absorbance was detected using a Sunrise ELISA reader (Tecan, Salzburg, Austria), with 450 nm as the primary wavelength.

### Statistical analysis

The data are presented as the mean and standard deviation (SD) unless stated otherwise. Data were analysed using a GraphPad Prism 6 (GraphPad Software, San Diego, CA, USA). The normal distribution was assessed by the D’Agostino and Pearson omnibus normality test. For the comparison between groups, the unpaired t-test or Mann-Whitney test were used. Pearson’s and Spearman’s correlation coefficients were calculated to assess the relationship between the CLU levels and other parameters. *P*-values less than 0.05 were considered statistically significant.

## Results

The patients and the control group did not differ in age, gender or CRP levels. However, the patients with erosive OA were older than those with non-erosive disease (*p* = 0.023). The CRP levels were comparable between both of the groups with hand OA. The AUSCAN total score and its subscale for pain were significantly higher in patients with erosive than in those with non-erosive OA (*p* = 0.048 and *p* = 0.032, respectively). Patients with erosive OA had more osteophytes (*p* = 0.003) and higher GS and PD synovitis total scores (*p* < 0.001 and *p* = 0.014, respectively) as well as higher number of affected joints (*p* < 0.001 and *p* = 0.009, respectively) compared to those with non-erosive disease (Table 1).

### Clusterin levels are lower in patients with hand OA

**Fig. 1:**
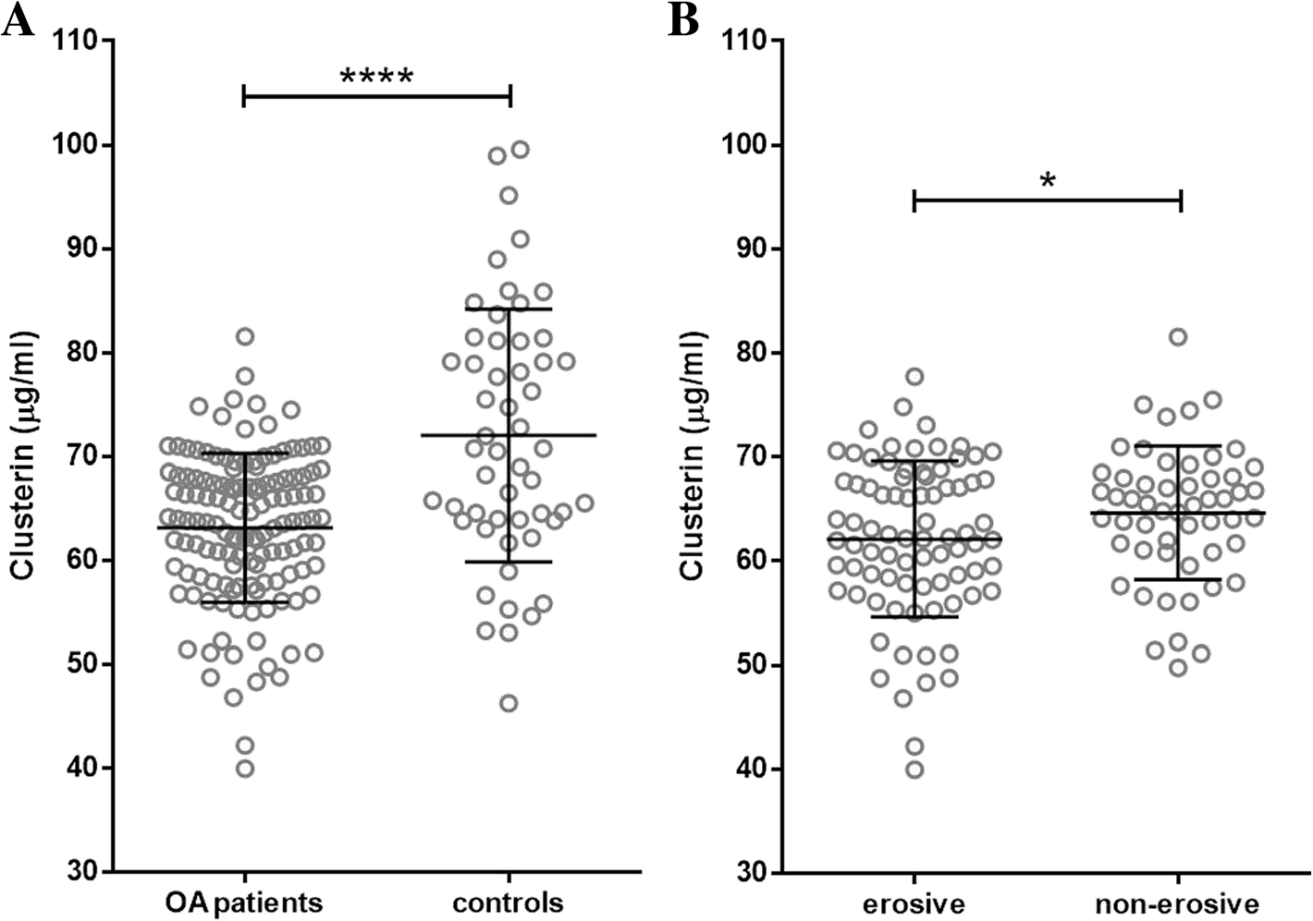
Serum levels of clusterin were significantly lower in patients with hand osteoarthritis (OA) compared to healthy controls (**a**), and in patients with erosive OA compared to those with non-erosive OA (**b**). (**** *p* < 0.0001; * *p* < 0.05)

The serum concentrations of CLU were significantly lower in the patients with hand OA than in the healthy subjects (63.12 ± 7.17 vs 72.02 ± 12.19 μg/ml; *p* < 0.0001) (Fig. 1a). After dividing the patients into disease subsets, the difference remained statistically significant for both erosive (*p* < 0.0001) and non-erosive (*p* < 0.0001) OA. Moreover, the patients with erosive disease had significantly lower CLU levels than those with non-erosive OA (62.11 ± 7.51 vs 64.64 ± 6.42 μg/ml; *p* = 0.044) (Fig. 1b). The CLU levels in hand OA patients were not affected by the concurrent presence of knee and/or hip OA (63.35 vs 62.95 μg/ml for knee OA, 62.98 vs 63.18 μg/ml for hip OA, 63.47 vs 63.03 μg/ml for knee and hip OA; *p* > 0.05 for all comparisons).

### Clusterin levels are inversely associated with pain in patients with erosive hand OA

**Fig. 2:**
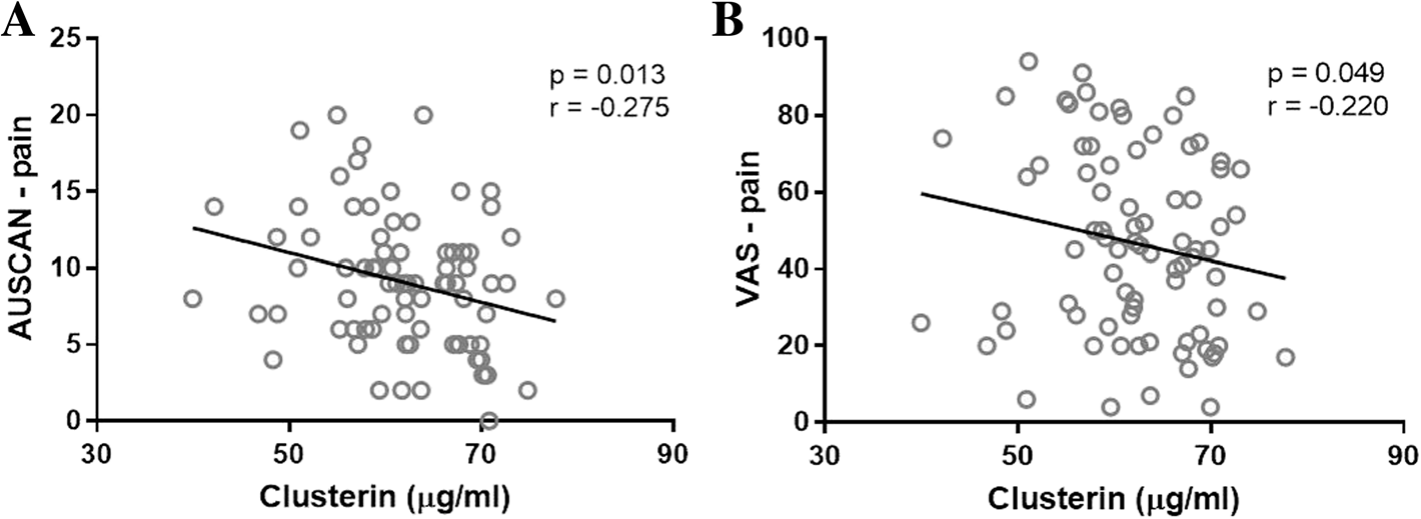
Serum levels of clusterin negatively correlated with the AUSCAN subscale score for pain (**a**) and the VAS for pain (**b**) in patients with erosive disease. (AUSCAN: Australian/Canadian, VAS: visual analogue scale)

Associations of CLU levels with clinical and laboratory parameters are shown in Table 2. There were no significant correlations between the CLU levels and the algofunctional index and total AUSCAN score in any of the patients with hand OA; however, among those with erosive disease, the CLU levels were negatively correlated with pain as assessed by the AUSCAN and the VAS (*r* = − 0.275; *p* = 0.013 and *r* = − 0.220; *p* = 0.049, respectively) (Fig. 2). The CLU concentrations were not associated with age, sex or body mass index. There were also no significant correlations between the CLU levels and disease duration, CRP levels, ultrasound-determined synovitis and osteophytes.

**Table 2:** Correlations between serum CLU levels, clinical and laboratory parameters

	OA patients	Erosive	Non-erosive
AUSCAN	r = -0.087	r = -0.151	r = 0.100
	p = 0.318	p = 0.179	p = 0.470
AUSCAN - pain	r = -0.166	r = -0.275	r = 0.071
	p = 0.054	p = 0.013	p = 0.610
AUSCAN - stiffness	r = -0.107	r = -0.124	r = -0.097
	p = 0.218	p = 0.271	p = 0.487
AUSCAN - function	r = -0.009	r = -0.066	r = 0.144
	p = 0.914	p = 0.558	p = 0.299
Algofunctional index	r = -0.139	r = -0.151	r = -0.075
	p = 0.107	p = 0.179	p = 0.590
VAS – pain , mm	r = -0.084	r = -0.220	r = 0.201
	p = 0.331	p = 0.049	p = 0.145
CRP, mg/l	r = 0.037	r = -0.004	r = 0.117
	p = 0.674	p = 0.969	p = 0.401
Disease duration, years	r = -0.032	r = -0.024	r = -0.011
	p = 0.713	p = 0.832	p = 0.938
US osteophytes, n	r = 0.156	r = 0.189	r = 0.214
	p = 0.072	p = 0.091	p = 0.120
GS synovitis (total)	r = 0.007	r = 0.042	r = 0.130
	p = 0.937	p = 0.711	p = 0.349
GS synovitis (joint count)	r = 0.001	r = 0.023	r = 0.133
	p = 0.990	p = 0.840	p = 0.339
PD synovitis (total)	r = 0.083	r = 0.111	r = 0.217
	p = 0.341	p = 0.324	p = 0.114
PD synovitis (joint count)	r = 0.079	r = 0.097	r = 0.236
	p = 0.362	p = 0.389	p = 0.085

*AUSCAN* Australian/Canadian, *CRP* C-reactive protein, *GS* gray scale, *OA* osteoarthritis, *PD* power Doppler, *US* ultrasound, *VAS* visual analogue scale

## Discussion

In this study, we found lower levels of CLU in patients with hand OA than in healthy subjects and an inverse association between CLU levels and pain in patients with erosive disease.

Erosive hand OA is a more severe form of hand OA than non-erosive OA, both clinically and radiographically, and is accompanied by worse pain and disability [19, 20]. In our study, we found significantly higher scores on the AUSCAN and its pain subscale in patients with erosive than in patients with non-erosive OA. Inflammatory signs were also reported to be more frequent in erosive than in non-erosive disease [21, 22]. In addition, Punzi et al. [23] found higher CRP levels in erosive OA patients than in those with non-erosive hand OA and suggested CRP as a potential marker of disease activity. However, no differences in the CRP levels between erosive and non-erosive OA were found in other studies [24, 25]. A number of other biomarkers have been studied in hand OA, including markers of inflammation [26]. Recently, Fioravanti et al. [27] reported significantly higher levels of serum myeloperoxidase (MPO) in patients with hand OA than in healthy controls. Moreover, patients with erosive OA showed significantly elevated MPO levels than those with non-erosive OA, which confirmed the results of a previous study [28] and further supported the involvement of inflammation in the pathogenesis of hand OA, especially in the erosive disease. In the present study, we observed higher ultrasound-determined synovitis total scores as well as the number of affected joints in patients with erosive OA compared to those with non-erosive disease. However, no significant differences in the CRP levels between the two subgroups of patients were found.

In this study, we found lower serum levels of CLU in the patients with hand OA than in the healthy controls. Several other studies have investigated CLU in OA [9–11]; however, this is the first study to explore the CLU levels in patients with hand OA. The expression of CLU mRNA has been reported to be higher in OA than in healthy cartilage [9, 10]. Fandridis et al. [10] also reported higher serum levels of CLU in patients with knee and hip OA than in healthy controls. The reason for these apparently contradictory results may be caused by the different locations of the affected joints. Nevertheless, we did not find any differences in the CLU levels between the hand OA patients with and without knee and/or hip OA. It is also important to note that, in our study, the samples obtained from the patients and the healthy subjects were analysed at the same time, whereas Fandridis et al. used the data from the healthy controls from their previous study and analysed only the patient group [10].

A previous study has shown that CLU mRNA expression in synovial tissue is lower in patients with RA than in patients with OA and healthy individuals [11]. We found significantly lower serum levels of CLU in patients with erosive OA compared with patients with non-erosive hand OA. Bone erosions are present in RA as well as in erosive OA, although their locations differ. The lower expression of CLU in these diseases may be explained by the potentially protective role of CLU against the development of bone erosions [13]. A recent study also suggests a protective function of CLU in inflammation and autoimmune diseases [29], which is in agreement with the study of Newkirk et al. [30] that reported lower serum concentrations of CLU in systemic lupus erythematosus and found negative correlations among CLU levels, disease activity and disease symptoms. In our study, we reported a negative correlation between the CLU levels and hand pain in patients with erosive OA. No such association was found in patients with non-erosive disease. Therefore, we can speculate that CLU may play a role in the pathology of erosive hand OA.

This study has several limitations. First, its design was cross-sectional. Therefore, a long-term prospective study is needed to further investigate the role of CLU in hand OA. Second, CLU levels might be affected due to the effects of other involved joints or diseases. However, we did not observe a difference in the CLU levels between the hand OA patients with and without radiographic hip and/or knee joint involvement.

## Conclusions

In conclusion, we demonstrate here for the first time that lower serum levels of CLU are found in patients with hand OA, especially in those with erosive disease, and that a negative association exists between CLU concentrations and hand pain. These data suggest a possible involvement of CLU in the pathophysiology of erosive hand OA and further support its role in the development of bone erosions.

## Abbreviations

ACR: American College of Rheumatology
AUSCAN: Australian/Canadian
BMI: Body mass index
CLU: Clusterin
CRP: C-reactive protein
ELISA: Enzyme-linked immunosorbent assay
FLS: Fibroblast-like synoviocytes
GS: Gray scale
nCLU: Nuclear clusterin
NF: Nuclear factor
OA: Osteoarthritis
PD: Power Doppler
RA: Rheumatoid arthritis
sCLU: Secretory clusterin
TNF: Tumor necrosis factor
US: Ultrasound
VAS: Visual analogue scale
